# Design and Evaluation of Losartan Transdermal Patch by Using Solid Microneedles as A Physical Permeation Enhancer

**DOI:** 10.22037/ijpr.2019.1100912

**Published:** 2020

**Authors:** Ericka Anguiano Almazan, Pablo Serrano Castañeda, Roberto Diaz Torres, Jose Juan Escobar-Chavez

**Affiliations:** a *Laboratorio 12: Sistemas Transdérmicos, Unidad de Investigación Multidisciplinaria, Departamento de Ingeniería y Tecnología, Facultad de Estudios Superiores Cuautitlán, Universidad Nacional Autónoma de México, Cuautitlán Izcalli, Estado de México, México. *; b *Laboratorio 9: Toxicología, Unidad de Investigación Multidisciplinaria, Departamento de Ingeniería y Tecnología, Facultad de Estudios Superiores Cuautitlán. Universidad Nacional Autónoma de México, Cuautitlán Izcalli, Estado de México, México.*

**Keywords:** Transdermal patch, Solid microneedles, Polyvinylpyrrolidone, Skin, Losartan; Eudragit E100®

## Abstract

The development of a losartan potassium patch for the treatment of hypertension showed that a combination of hydrophobic and hydrophilic polymers, using as a plasticizer citroflex and succinic acid as a cohesion promoter result in homogeneous films. The effect of the Eudragit^®^ E100, PVP K30, citroflex and succinic acid in the bioadhesion, postwetting bioadhesion, resistance to rupture and drug release, was studied. The succinic acid in synergy with the plasticizer (citroflex) modifies the characteristics of the polymeric matrix of Eudragit^®^ E100, increasing the release and the resistance to rupture of transdermal patches. For the case of the hydrophilic polymer PVP K30, it increases the bioadhesion and drug release by creating porous matrices. From a previous experimental design, the optimal formulation was acquired, and this formulation was physicochemically characterized. A transdermal patch was obtained with the next dimensions and characteristics: 28.46 ± 0.055 mm in diameter and 0.430 ± 0.008 mm in thickness, a bioadhesion of 1063.05 ± 60.33 gf, postwetting bioadhesion 995.9 ± 72.53 gf significantly decreased. The breaking strength was of 1301.5 ± 96.5 gf, surface pH patch of 6, constriction of 0% at 7 days, and 94.0366 ± 1.8617% of losartan content. The 93% of the drug is released at 4 h (n = 6), adjusting to the kinetic model of Higuchi and Peppas. In the *in-vitro* penetration studies by passive diffusion, a flow (J) of 42.2 μg/cm^2^h, a permeability constant (kp) of 2.1793E-03 cm/h and a latency time (t_L_) of 17.20 h and with the use of microneedles a flow (J) of 61.7 μg/cm^2^h, a permeability constant (kp) of 3.1869E-03 cm/h and a latency time (t_L_) of 17.74 h were obtained.

## Introduction

Hypertension is defined as a systolic blood pressure that remains above 140 mm Hg or a diastolic pressure that remains above 90 mm Hg. Blood pressure is the force exerted by the blood against the walls of the arteries when being pumped by the heart and when the blood pressure is higher; more effort must be made by the heart to pump blood. Normal blood pressure in adults is 120 mm Hg when the heart beats (systolic tension) and 80 mm Hg when the heart relaxes (diastolic tension) (1-3). Among antihypertensive drugs, losartan potassium (LP) (angiotensin II receptor blocker) can be used as a first-line agent to treat hypertension without complications, hypertension in people with diabetes, heart failure, nephropathy, and left ventricular hypertrophy. It can also be used as a second-line agent in the treatment of congestive heart failure, systolic dysfunction, myocardial infarction, and coronary artery disease in those intolerant to angiotensin-converting enzyme inhibitors (4).

The transdermal or percutaneous route can be considered for drugs with adequate physicochemical and pharmacological char-acteristics as an alternative to oral route since this route of administration allows a controlled and sustained release of the drug (5). These transdermal systems or transdermal patches (TP) release the drug at a constant speed over a prolonged period (days), which allows the plasma concentration of the drug to remain within the therapeutic range (6). 

Among the main advantages offered by a transdermal patch are: the elimination of the first hepatic passage, the avoidance of multiple doses and digestive problems associated with oral administration and the obtention of constant plasma levels of the drug that result in a better attachment to the pharmacological treatment (5, 7-11).

The use of physical enhancers like microneedles have shown good results to increase the delivery of the drugs through the skin (12). The use of microneedles has very important advantages for transdermal drug delivery, for example, they are painless, easy to use and safe (12-14), they increased time interval of drug activity, dose, and adverse reactions reduction and facility to remove the system instantly. In this case, the microneedles have the intention to enhance the drug delivery of losartan.

Due to the advantages presented by a transdermal system, a TP was developed in order to allow the administration of LP for the treatment of hypertension and its complications, where the components of the matrix system permit a controlled drug release. This developed matrix system has the purpose of generating a new pharmaceutical form that increases the bioavailability of LP and avoids multiple doses to improve therapy.

## Experimental

*Materials*

We used analytical grade reagents that comply with Analytical Chemistry Society (ACS) specifications and were as follows: Losartan potassium (Gylsa Group México), Eudragit^®^ E100 (HELM de México), polyvinylpyrrolidone K30 (Droguería Cosmopolita), methanol (J.T Baker), citroflex (Sigma-Aldrich), succinic acid (Sigma-Aldrich), dibasic sodium phosphate (Fermont), sodium hydroxide (MEYER), 4-(2-hydroxyethyl)-1-piperazineethanesulfonic acid (HEPES) buffer (Sigma-Aldrich), and Milli-Q quality distilled water (Millipore Inc.). 

*Transdermal patches preparation*

An appropriate amount of Eudragit E100^®^ was added in methanol until dissolution and then the rest of the ingredients were added for each formulation. The films obtained were dried at room temperature for 48 h with an area of 283.53 mm^2 ^(Table 1). We use a multilevel factorial design (18 formulations) to find the optimal formulation; the software used was Statgraphics Centurion XV. II (Table 2). We analyzed 6 formulations, from this factorial design and we obtained optimal formulation. These formulations were characterized by the following tests: tensile strength, bioadhesion test, post wetting-bioadhesion and drug release test. The optimal formulation was characterized by uniformity of drug content, superficial pH, the percentage of constriction, tensile strength, bioadhesion test and post wetting-bioadhesion, drug release test, and *in-vitro* percutaneous absorption studies. The variables were the percentages of succinic acid and citroflex used.

*Tensile strength*

This test was performed using the texturometer (Brookfield CT3 Texture Analyzer, USA with TexturePro CT Software) that has a load cell of 4500 g. To perform this test, 10 patches with an area of 8.4 cm^2^ were held with tweezers in the lower and upper part of the texturometer. For this test a pre-test speed of 2 mm/s was used, a test speed of 0.5 mm/s. A tension force of 6.8 g and a maximum separation distance of 100 mm were used, determining the force at which the patch breaks (11, 15-17).

*Bioadhesion test and post wetting-bioadhesion*

The studies were carried out using a texture analyzer (Brookfield model CT3 Texture analyzer, USA with TexturePro CT Software). The skin samples obtained from abdominoplasty were donated by the Hospital Angeles Inn Chapultepec, México CDMX, they were placed at the bottom of the texturometer, using a cylindrical probe (perplex cylinder 1.27 cm^2^) and TP (0.95 cm^2^). The conditions for the test were: pre-test velocity of 2 mm/s, load force of 6.8 gf and a speed of 0.5 mm/s (16). All experiments were performed in triplicate for each formulation.

The post wetting-bioadhesion is like the bioadhesion, but the difference lies in a pre-hydration of the transdermal patch with an atomizer at a distance of 30 cm with deionized water 10 min before performing the test.

*Drug release study*

The studies were made in the equipment for dissolution studies (MAYASA model APPM-0250, México) with apparatus number 5 USP (paddle over disc method) for assessing drug release from the prepared patches. The conditions were 500 mL phosphate buffer pH 5.5 referring to physiological skin pH (17), at 37.5 °C with stir (50 rpm). Samples of 3 mL were taken at times of 5, 10, 15, 20, 30 min and 1, 2, 3, 4 h, without replacement of the medium. Subsequently, the amount of drug released as a function of time was quantified by spectrophotometry at 248 nm (15-19).

*Uniformity of drug content*

Ten transdermal patches (TP) were weighed and then cut in the circles with an area of 283.53 mm^2^, each of the circles was weighed and the theoretical amount of losartan potassium was calculated. Each of the samples was dissolved in 20 mL of methanol to extract the drug from the polymer matrix, and the dilutions were made in distilled water (2:25, 1:10 mL). Finally, the absorbance of each of the samples was measured at 248 nm in a UV-Vis spectrophotometry (Velab model VE-5100UV, USA) (Table 1) (16, 20 and 21). The method was previously validated, complied with the specifications as linearity parameters (coefficient of determination r^2 ^**˃ **0.99, slope coefficient CV ˂ 2%), accuracy and repeatability (coefficient of variation of replicates CV ˂ 2%).

*Superficial pH*

For each of the formulation, 500 μL of distilled water was added to the surface of each patch (10 determinations), after waiting 2 min, the pH of the surface was determined with reactive strips.

*Percentage of Constriction*

Patches with an initial area of 283.53 mm^2^ were used for the test. The diameter of transdermal patches was measured at the initial time, 30 min and at 7 days after cutting. The patches were exposed to the environment and the percentage of constriction was calculated using the following Equation: 

Constriction% = (D_1_ - D_2_/D_2_) × 100

Where: D_1_ is the initial measurement and D_2_ the final measurement. The result obtained corresponds to the percentage of constriction (22).

*In-vitro percutaneous absorption studies*

The studies were performed using vertical Franz type cells. As a membrane between the two compartments, the abdominal human skin was used. The optimal formulation was placed on the skin. Receiver compartment was filled with a buffer solution of (HEPES) at pH 7.4. The assembly of the cells was placed on a magnetic stirrer with temperature control. The receiver solution shared with a magnetic bar and a thermostated at 32 °C. The sampling was performed at different intervals for 36 h and the drug content was determined by UV-Vis spectrophotometry at 248 nm, the method was previously validated, complied with the specifications for linearity parameters (coefficient of determination r^2 ^˃ 0.99, slope coefficient CV ˂ 2%), accuracy, and repeatability (coefficient of variation of replicates CV ˂ 2%). Cumulative drug accumulations per square centimeter of the formulations were graphed as a function of time (11, 15 and 16). We compare the passive diffusion of losartán potassium and the permeation of it with the use of microneedles (MT Dermaroller 2.25 mm approved for the Food Drug Administration), the MT Dermaroller was passed 15-20 times in horizontal, vertical, and oblique directions on the skin for percutaneous absorption studies.

**Figure 1:**
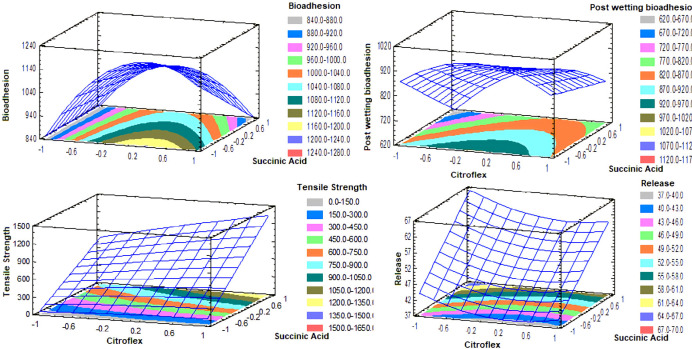
Response surface graphics estimated for each of the tests performed

**Figure 2:**
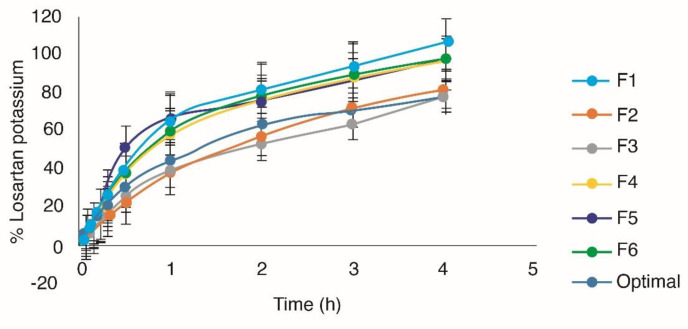
Release profiles of the evaluated formulations including the optimal formulation

**Figure 3:**
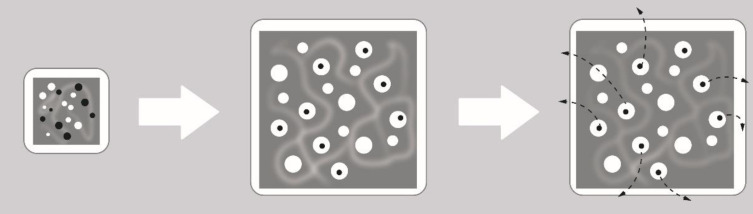
Diffusion release mechanism of drug through the polymeric matrix (31).

**Figure 4:**
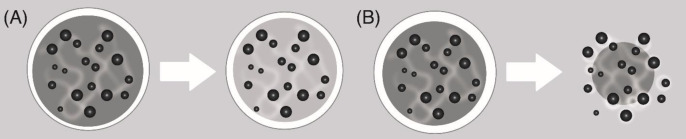
(A) Mass erosion and (B) Surface erosion (31)

**Figure 5:**
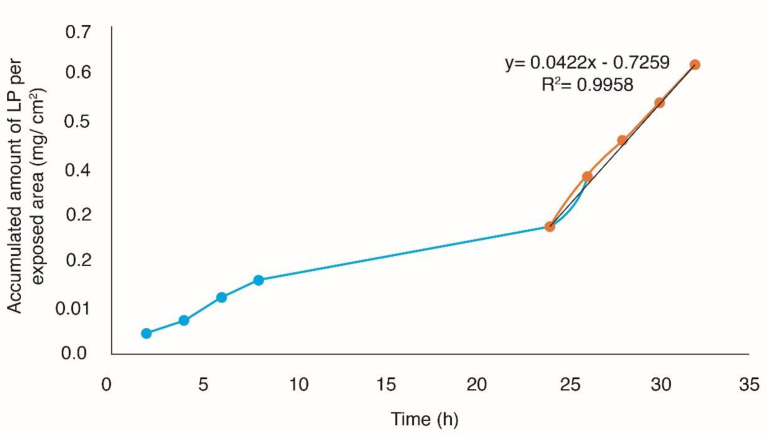
Permeation profile through human skin by passive diffusion

**Figure 6:**
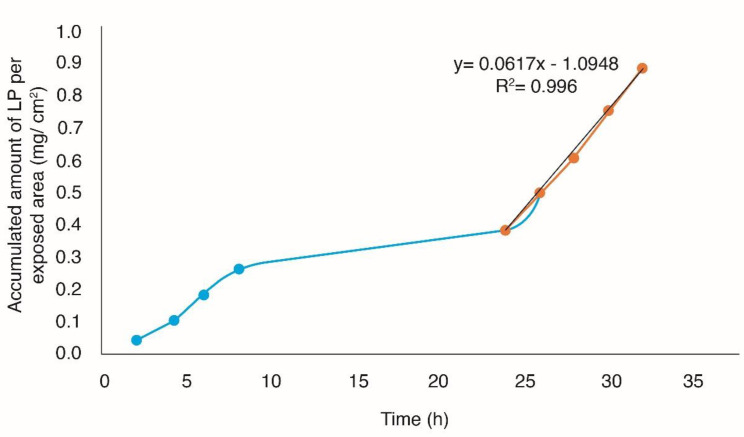
Permeation profile of losartan through human skin using solid microneedles as a physical enhancer

**Table 1:** Components of each transdermal patch

**Formulation**	**Succinic Acid** **(mg)**	**Citroflex** **(µL)**	**Eudragit** ^®^ ** E100** **(mg)**	**PVP K30** **(mg)**	**Losartan Potassium** **(mg)**	**Methanol** **(mL)**
F1	0	800	1000	1000	1000	25
F2	300	800	1000	1000	1000	25
F3	300	750	1000	1000	1000	25
F4	0	700	1000	1000	1000	25
F5	0	750	1000	1000	1000	25
F6	300	700	1000	1000	1000	25
Optimal	300	765	1000	1000	1000	25

**Table 2:** Formulations of the experimental design

**Formulation**	**A** ^*^	**C** ^*^	**A (mg)**	**C (µL)**
F1	-1	1	0	800
F2	1	1	300	800
F3	1	0	300	750
F4	-1	-1	0	700
F5	-1	0	0	750
F6	1	-1	300	700

F: formulation; A^*^: coded levels of succinic acid; C^*^: coded levels of citroflex; A: uncoded levels of succinic acid; C: uncoded levels of citroflex.

**Table 3:** Average results of bioadhesion, post wetting bioadhesion, tensile strength and drug release

**Formulation**	**Bioadhesion ** **(gf)**	**Post wetting bioadhesion (gf)**	**Tensile strength (gf)**	**Release (%)**
F1	1111.83 ± 258.70	596.00 ± 372.01	57.83 ± 100.17	51.81 ± 18.83
F2	970.17 ± 408.33	742.33 ± 92.07	1013.83 ± 891.37	58.64 ± 7.91
F3	1045.17 ± 538.98	903.50 ± 217.84	1372.50 ± 1112.65	60.06 ± 17.53
F4	964.17 ± 193.24	1097.67 ± 642.11	179.50 ± 310.90	54.89 ± 3.33
F5	1008.50 ± 375.79	848.00 ± 418.82	844.50 ± 1237.49	46.39 ± 18.71
F6	783.83 ± 267.86	751.50 ± 128.56	911.90 ± 1508.71	36.08 ± 9.71
Optimal	1063.05 ± 60.33	995.9 ± 72.53	1301.5 ± 96.54	93.11% ± 2.11

**Table 4:** Kinetic results of the release of the evaluated formulations

**Formulation**	**Order Zero**		**Order One**		**Higuchi**		**Korsmeyer-peppas**		
	$r^{2}$	$k_{0}$ ** (** $h^{-1}$ **)**	$r^{2}$	$k_{1}$ ** (** $h^{-1}$ **)**	$r^{2}$	$k_{H}$ **(** $h^{-1/2}$ **)**	$r^{2}$	**n**	$k_{\mathrm{KP}}$ **(** $h^{-n}$ **)**
F1	0.9187	34.453	0.6579	0.2407	0.9868	82.133	0.9939	1.1092	2.1203
F2	0.9822	37.256	0.7586	0.2873	0.9963	86.309	0.9758	0.9138	1.8274
F3	0.9583	33.091	0.7182	0.2627	0.9982	77.686	0.9956	1.0340	1.9602
F4	0.9244	43.878	0.6365	0.2749	0.9900	104.45	0.9999	1.4112	2.3181
F5	0.8599	43.707	0.6030	0.2596	0.9539	105.88	0.9950	1.2674	2.2584
F6	0.9013	44.628	0.6276	0.2991	0.9786	106.96	0.9997	1.4518	2.2542
Optimal	0.911	44.685	0.6200	0.3360	0.9810	106.97	0.9990	1.6030	2.3490

F1: succinic acid 0 mg, citroflex 700 μL; F2: 0 mg succinic acid, 750 μL citroflex; F3: succinic acid 0 mg, citroflex 800 μL; F4: succinic acid 300 mg, citroflex 700 μL; F5: succinic acid 300 mg, citroflex 750 μL and F6: succinic acid 300 mg, citroflex 800 μL.

**Table 5:** Dimensions of Optimal TP

Optimal	Dimensions	
	Diameter (mm)	Thickness (mm)
**1**	28.55	**0.44**
**2**	28.48	**0.43**
**3**	28.36	**0.44**
**4**	28.50	**0.43**
**5**	28.45	**0.43**
**6**	28.42	**0.43**
**7**	28.43	**0.42**
**8**	28.52	**0.42**
**9**	28.49	**0.42**
**10**	28.48	**0.44**
**x**	28.468	**0.430**
**S**	0.055	**0.008**

**Table 6:** Constriction percentage at the initial time 30 min and 7 days

	**Initial time**	**30 min**		**7 days**	
**Optimal TP**	**Diameter (mm)**	**Diameter (mm)**	**Constriction (%)**	**Diameter (mm)**	**Constriction (%)**
1	19.63	19.63	0	19.63	0
2	18.68	18.68	0	18.68	0
3	19.42	19.42	0	19.42	0
4	19.39	19.39	0	19.39	0
5	19.28	19.28	0	19.28	0
6	19.31	19.31	0	19.31	0
7	19.08	19.08	0	19.08	0
8	19.27	19.27	0	19.27	0
9	19.71	19.71	0	19.71	0
10	19.24	19.24	0	19.24	0
x	19.301	19.301	0	19.301	0
S	0.286	0.286	0	0.286	0

**Table 7:** Results of uniformity of drug content

**Optimal** **TP**	**Weight of TP** **(g)**	**Amount of LP** **(g)**	**Sample weight** **(g)**	**Theoretical quantity LP** **(mg)**	**Abs**	**Real Amount LP** **(mg)**	**LP** **(%)**
1	4.1805	1.0842	0.1175	30.4733	0.354	28.3333	92.9777
3	4.1805	1.0842	0.1152	29.8768	0.351	28.0952	94.0371
4	4.1575	1.0644	0.1153	29.519	0.34	27.2222	92.2193
5	4.1575	1.0644	0.117	29.9543	0.35	28.0159	93.5289
6	4.1575	1.0644	0.1205	30.8503	0.36	28.8095	93.3848
8	4.0815	1.055	0.1199	30.9922	0.36	28.8095	92.9575
9	4.0815	1.055	0.1095	28.3039	0.339	27.1429	95.8978
10	4.0608	1.0226	0.1108	27.9019	0.342	27.381	98.1329
11	4.0608	1.0226	0.1117	28.1286	0.334	26.746	95.085
12	4.0608	1.0226	0.118	29.715	0.342	27.381	92.1451
						x	94.0366
						S	1.8617
						C.V%	1.9797

## Results and Discussion

The composition of all transdermal patches evaluated is shown in Tables 1 and 2 coded levels. 

*Bioadhesion*

The lack of adhesion of the transdermal systems to the skin is a critical factor directly related to the therapeutic effect. For the process of penetration of the drug, a complete contact of the TP on the skin during the entire period of application is essential. If the TP is totally or partially separating the effective area and therefore the absorption of the drug changes unpredictably, this can lead to a therapeutic failure. Therefore, the adhesive must ensure initial adhesion but must have enough cohesive strength to remove it cleanly, leaving no residue (23, 24). The results of bioadhesion for each transdermal losartan patch of the design can be seen in Table 3 and Figure 1.

When the Eudragit E100^®^ patch is applied to the skin, it is expected that this polymer will adhere to skin for its lipophilic character. In addition, its adhesive properties increase when a plasticizer (Citroflex) is added (Table 3) (25).

*Post wetting-bioadhesion*

Post-wetting bioadhesion has the same importance as bioadhesion, except that this test considers wetting of the TP by transpiration or by external conditions such as environmental and washing. The results obtained from the design are shown in Table 3. 

In the design, none of the factors has a significant effect (*p *> 0.05). Succinic acid has a linear tendency, while citroflex has a quadratic tendency, that is, at high and low levels, the minimum response is obtained. In the levels -0.2-0.2 of citroflex, and not adding succinic acid, an estimated maximum response of 970-1020 gf is obtained (Figure 1). The results (Table 3) presented a normal distribution, so we proceeded to perform a *t*-Student test (*p* < 0.05) and compared (bioadhesion and bioadhesive post-wetting), indicating that the matrix system once wetted is less bioadhesive. This is because when moisturizing the skin decreases its lipophilic character and does not allow the Eudragit E100^®^ matrix to adhere properly.

*Tensile strength*

The importance of this test is if the TP does not have a good resistance to rupture, there may be problems of safety, therapeutic efficacy and they have not supplied the adequate dose to the patient (16, 17). The results obtained are found in Table 3.

The properties of the polymer matrix can be modified using cohesion enhancers (crosslinkers) with free carboxyl groups; such compounds enter an ionic interaction with the tertiary amine functional groups of Eudragit^®^ E100. The mechanical properties of the Eudragit^®^ E100 are improved using a plasticizer; however, the action of the plasticizer and the cross-linking of the succinic acid together increase the mechanical properties of the films with Eudragit^®^ E100. The succinic acid allows the crosslinking of the polymer chains in “layers” that are “slid” over one another by the action of the plasticizer, this crosslinking allows a greater resistance to rupture than the chain of a polymer uncrosslinked (26). The succinic acid has a significant effect (*p* < 0.05) and positively affects the response (Figure 1). To obtain an estimated maximum response it is necessary to use levels 0.4-1 of citroflex and 0.8-1 of succinic acid. On the other hand, the incorporation of PVP K30 that is a hydrophilic polymer makes the film elastic, smooth and flexible. This parameter is of vital importance for the ideal manipulation of the transdermal system, either during its evaluation or during its application. Very fragile films could modify their surface easily resulting in a therapeutic ineffectiveness and/or complicating their manipulation (25, 27 and 28).

*Drug release test*

The importance of the test is to predict the rate and duration of drug release and ensure the constant release of drug from the polymer matrix of TP (16, 29).

In the design, succinic acid positively affects the response (*p* < 0.05). The citroflex has a quadratic tendency and the succinic acid a linear trend. In the levels -0.3 to -1 of citroflex, and 0.8-1 of succinic acid, an estimated maximum response of 64-67% of drug released per hour was obtained (Figure 1). The incorporation of crosslinker in the Eudragit E100^®^ matrix is one of the most used methods to modify the release of the drug, generally, the effect of the cohesion promoter on the release of drug from polymeric matrix is based on its influence on the disposition of polymer within the matrix (16). In all the formulations, a release of more than 70% of the drug was obtained before 3 h (Figure 2).

In order to determine the kinetics of drug release in the different formulations, the data were adjusted to the zero order, first order, Higuchi, and Korsmeyer-peppas models. The results can be seen in Table 4.

The results of Table 4 indicate that from F1 to F4 the release mechanism occurs both by diffusion and erosion, for F5 and F6 the mechanism of release is controlled by relaxation-erosion (30). Finally, the optimal formulation was obtained to maximize all the answers (Table 1). For the optimal formulation, it is important that the drug is released in at least 80% of the matrix in order to ensure that once placed on the skin, the drug can be released from the polymeric matrix to the stratum corneum. As shown in Figure 2, 93.11% ± 2.11 of losartan is released at 4 h, which indicates that the drug is available for absorption through the skin. The optimal formulation conforms to the kinetic model of Higuchi and Korsmeyer-peppas (Table 4). Therefore, the release of the drug follows a diffusion and erosion mechanism.

The term “diffusion” refers to the actions of drug molecules after exposure to stimuli that affect their external environment (Figure 3). The rate at which water can swell the matrix of a cross-linked system is significantly faster than the rates of degradation or dissolution, given by the erosion mechanism. In matrix systems the permeation of the dissolution medium leads to swelling systems, since the matrix is composed of both polymer and drug molecules, the swelling effect is seen as a uniform volume expansion of the bulk polymer material, by causing the opening of pores along the matrix structure, for efficient diffusion of drug molecules to occur, the pore size of the swollen matrix must greatly exceed the size of the drug molecule.

The mechanism of erosion is associated with changes in the physicochemical properties of the polymeric material, physical processes such as swelling, deformation or structural disintegration, weight loss and eventual loss of functions. The speed limitation stage of erosion-controlled release systems is dissolution. There are two types of erosion (30, 31): a) Mass erosion (Figure 4A): In the case of mass erosion, the polymer degrades or dissolves uniformly throughout the volume of the polymer system. As the degradation proceeds, the volume of the polymeric material remains constant while the mass of the polymeric material is reduced, resulting in a decrease in the density of the degrading polymer. The transdermal patches evaluated in this study present mass erosion. b) Surface erosion (Figure 4B): The polymeric material is degraded from the outer surface to the interior uniformly only at the interface between most of the material and the surrounding environment. As the degradation progresses, the volume of the material decreases linearly with the mass, so that the density of the material remains constant (30).

The films made with Eudragit E100^®^ have a low release rate due to Eudragit hydrophobicity which restricts the release of the drug from the polymeric matrix. However, the plasticizer acts relaxing the polymer network which allows a diffusion of the drug through the matrix. On the other hand, the incorporation of a hydrophilic polymer in this case PVP K30 increases the release, since this in contact with the dissolution medium creates pores through which the drug is released by the diffusion process. Regarding the erosion process, it is attributed to PVP K30 due to its high solubility in aqueous media (25, 32).

For the optimal transdermal patch, the following tests were performed: dimensions, the percentage of constriction, surface pH of TP, and uniformity of drug content.

*Dimensions*

The thickness and diameter are properties that must be considered in the design and development of a TP since they are directly related to comfort; they are more approved by the patient for their comfort and discretion if they have a small size and thickness. The results are in Table 5, obtaining an average diameter of 28.468 ± 0.055 mm and an average thickness of 0.430 ± 0.008, the dimensions of the patches turned out to be uniform with a minimum variation between them. The thickness is an indication of the homogeneous distribution of the components of the formulation on the molding surface, which is why they are important during their physicochemical characterization (33, 34).

*Constriction percentage*

The constriction test of the TP was performed at the initial time 30 min and 7 days. It is important that the TP does not present constriction, and the optimal formulation does not present constriction (*p* < 0.05), because it would lead to a variation of the area of the patch and it may also imply that there are irregularities in the surface, which would decrease the effective area of contact affecting directly the dosage of the drug. Table 6 shows the Optimal TP that has 0% constriction at the initial time 30 min and 7 days, which guarantees that the transdermal patches will maintain a smooth and uniform surface once placed on the skin (35).

*Superficial pH of transdermal patch*

The acidic or alkaline pH can cause skin irritation; it can affect the absorption of the drug if one of the characteristics of the drug for transdermal penetration is that it must be in its non-ionized form. Consequently, the surface pH of the patches was determined. The surface pH of all the samples evaluated (n = 10) had a value of 6, therefore, at this pH, it will not cause skin irritation (34).

*Uniformity of drug content*

The evaluation of this parameter is important to ensure that the transdermal patch contains the dose required to exert the desired therapeutic effect. The Mexican Pharmacopeia establishes a content of not less than 85.0% and not greater than 115% for transdermal systems and no unit should be outside the range of 75.0 to 125.0%. The relative standard deviation must be less than or equal to 6.0%. Table 7 shows the results obtained, the chemical content of the transdermal patches was 94.0366 ± 1.8617%, none of the patches evaluated is outside the range of 75.0 to 125.0% and the C.V% is less than 6.0%. The TP meet the acceptance criteria (36).

*In-vitro studies of percutaneous absorption*

*In-vitro* studies performed adequately have shown that they can provide a good prediction of percutaneous absorption *in-vivo*. Given this, the use of human skin is paramount, since it allows to provide real conditions to the experimentation because it is viable even after its extirpation (37). The accumulated amount of Losartan potassium per exposed area (mg/cm^2^) was plotted as a function of time, in order to obtain the profiles of permeation through the skin (Figures 5 and 6), obtaining the flow parameters (J), the permeability coefficient (kp) and latency time t_L_ (Passive diffusion K_p _= 2.1793E-03 cm/h, t_L _= 17.20 h, *J* = 42.2 µg/cm^2^h with Microneedles K_p _= 3.1869E-03 cm/h, t_L _= 17.74 h, *J *= 61.7 µg/cm^2^h). 

From the permeation parameters, it was determined that the optimal TP patch without the use of solid microneedles (passive diffusion) with an area of 24.68 cm^2^ releases a dose of 25 mg losartan potassium for ≈9 days and using microneedles with a patch with an area of 16.88 cm^2^ releases a dose of 25 mg losartan potassium for ≈6 days. 

Added to the fact that losartan would pass through the skin on the base of permeation studies, it is worth mentioning that losartan is metabolized into a 5-carboxylic acid derivative (E-3174) through an intermediate aldehyde (E-3179) mainly by cytochrome P450 (CYP2C9 and CYP3A4). E-3174 is an active metabolite with a potency of 10 to 40 times greater than its original compound, Losartan. Approximately, 14% of Losartan becomes E-3174; however, it was found that the AUC of E-3174 was 4 to 8 times higher than Losartan and E-3174 is considered as the main contributor to pharmacological effects. The expression of many cytochrome P-450 isoenzymes (CYP) in different types of skin cells has been recently described in the skin: Langerhans cells, keratinocytes, fibroblasts, and melanocytes. The epidermal activity of CYP in the skin is around of 2-4% compared to that of the liver, so the dose may possibly be decreased, since a high percentage of Losartan will not be metabolized compared to the oral route, in addition to that Losartan will be taken as such and this will also produce pharmacological effect (4, 16).

Microneedles of 2.25 mm in length were used because they have an important role in percutaneous absorption. In previous studies by Serrano *et al.* (2013), it was demonstrated that the 2.25 mm solid microneedles present greater penetration of the drug (15). This is because the 2.25 mm microneedles perfectly penetrate the stratum corneum generating disruption in the skin, making the drug can enter into the dermis (37).

Passive diffusion and the use of microneedles were compared using a *t*-student test, with no statistically significant difference (*p* > 0.05). The process of percutaneous absorption is conditioned by the lipophilicity of the drug, which can be expressed by its partition coefficient lipid/water. Losartan potassium has a Log P of 6.1 so it confers a lipophilic character, for that it does not require an enhancer of the skin penetration (4, 38).

## Conclusion

From the parameters of permeation using microneedles, it was determined that the optimal patch with an area of 16.88 cm^2^ releases a dose of 25 mg losartan potassium for ≈6 days; however, as there is no significant difference between the use of microneedles and passive diffusion, it is feasible to use the patch size of 22 cm^2^ by passive diffusion with duration of ≈9 days, which provides an effective therapy. With these results, the use of multiple doses, adverse effects associated with the dose and gastric irritation is avoided, making these systems more comfortable for the patient and improving adherence to treatment. In addition to that this TP would be less expensive since it would not require the use of a penetration enhancer.
